# Abnormal keratin expression pattern in prurigo nodularis epidermis

**DOI:** 10.1002/ski2.75

**Published:** 2021-12-01

**Authors:** L. L. Yang, B. Jiang, S. H. Chen, H. Y. Liu, T. T. Chen, L. H. Huang, M. Yang, J. Ding, J. J. He, J. J. Li, B. Yu

**Affiliations:** ^1^ Department of Dermatology Peking University Shenzhen Hospital Shenzhen Guangdong China; ^2^ Huzhou Center Hospital Huzhou China; ^3^ Department of Dermatology Affiliated Shenzhen Longhua People's Hospital of Southern Medical University Shenzhen Guangdong China; ^4^ Guanghe Hui Shenzhen Guangdong China; ^5^ National Cancer Center/National Clinical Research Center for Cancer/Cancer Hospital & Shenzhen Hospital Chinese Academy of Medical Sciences and Peking Union Medical College Shenzhen Guangdong China; ^6^ Department of Dermatology Shenzhen Baoan Maternal and Child Health Hospital Shenzhen Guangdong China; ^7^ Department of Plastic and Cosmetic Surgery Peking University Shenzhen Hospital Shenzhen Guangzhou China

## Abstract

**Background:**

Prurigo nodularis (PN) is a highly pruritic, chronic dermatosis and difficult to treat. PN lesions are characterized by existence of many hyperkeratotic, erosive papules and nodules. However, the pathogenesis of PN still remains unelucidated.

**Aim:**

To clarify the keratin role in the epidermis hyperproliferation, the keratin expression pattern in the PN lesional skin.

**Methods:**

In this study, we enrolled 24 patients with PN and 9 healthy control volunteers. K1/K10, K5/K14, K6/K16/K17 expression pattern were investigated by using immunohistochemical staining.

**Results:**

The lesional skin consists of the thickened spinous layers, in which active cell division was found. K5/K14 were upregulated in PN lesional epidermis, the staining signal localized in the basal layer and lower suprabasal layers. Hyperproliferation‐associated K6 was found in all layers of epidermal lesional skin, especially in the spinous layers. In contrast, K16 was only detected in the basal and lower suprabasal layers, K17 was observed in the basal and spinous layers. Terminal differential keratins K1/K10 were upregulated, detected in the pan‐epidermis, but spared in the basal and low suprabasal layers.

**Conclusion:**

The keratinocytes enter an alternative differentiation pathway, which are responsible for the activated keratinocyte phenotype, abnormal keratins expression potentially contributes to the keratinocytes proliferation, subsequently lead to increased lesional skin epidermis thickness, hyperkeratiosis and alteration of skin barrier properties.

## Graphical abstract

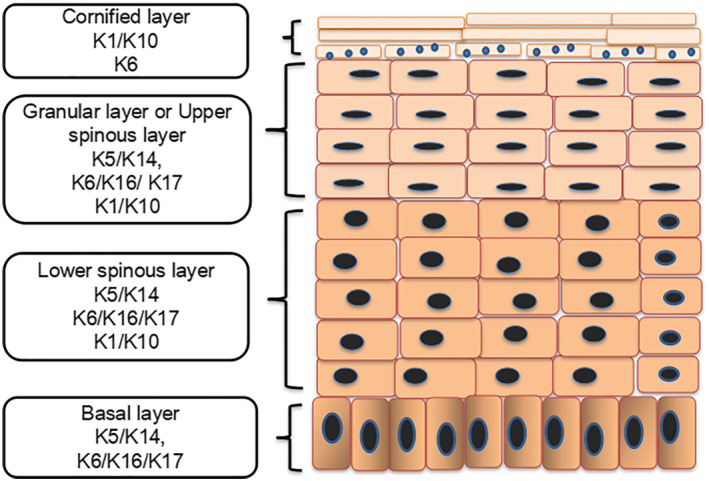

Figure 1. Keratins expression pattern in PN lesional skin epidermis

1

**What is already know about this topic?**

- Epidermal hyperplasia, inflammation and neurohyperplasia in the Prurigo nodularis (PN) dermis affect the formation and chronicity of PN.
- Keratin expression alterations in the epidermis layers forming specific patterns, which is related to the significant modulation upon epidermal diseases.
- Active keratin K6, K16 and K17 mRNA levels are increased in PN lesional skin.

**What does the study add?**

- High expression level of K5/K14 and K6 et al. correspond to the increased cell mitotic activity within the basal and suprabasal layers. Terminal differential keratins K1/K10 were upregulated in the suprabasal layers.
- Hyperproliferative and aberrant differentiation of Keratinocytes are involved in the lesional epidermis hyperplasia and hyperkeratiosis.

## 2 INTRODUCTION

Prurigo nodularis (PN) is defined by chronic pruritus dermatosis, multiple, firm nodules spread on the truck and the extensor aspects of the limbs.^1^, ^2^ Kwon et al. suggest a standardized diagnostic evaluation for PN including: presence of pruritus >6 weeks, ≥10 nodules ranging from 0.5–2.0 cm and on at least two different body surface areas.^3^ PN patients suffer from high itch intensity without effective treatment options, an itch‐scratch cycle results in a reduced quality of life.^4^, ^5^, ^6^ Therapeutically, IFSI‐guideline on prurigo nodularis was provided in 2020, it is advised to adopt a multimode approach, control itch and treat the pruriginous lesions et al.^7^

Histological features of PN epidermis showed compact orthohyperkeratosis (84%), irregular elongation of the rete ridges (58%), focal or broad hypergranulosis (52%),^6^ many types of skin cell altered, especially Keratinocytes (KC), dermal nerve fibres density increased,^1^, ^4^ pruritogenic mediators (neurokinin 1 receptor(NK1R), IL31RA, NGFR, et al.) expression patterns in KC underline abnormal KC differentiation in the PN pathogenesis.^8^, ^9^

The processes of activation and de‐activation KC require the regulatory changes in keratin gene expression. The keratins are fundamental components of the KC cytoskeleton, they are typical intermediate filament proteins, together with microfilaments and microtubules, which provide the structural resilience of epithelia cell.^10^, ^11^ The keratins are subdivided into two distinct classes according to sequence homology, type I keratin (acidic) (K9‐K40) and type II keratin (neutral/basic) (K1‐K8, K71‐K86).^10^, ^12^ Keratins form obligate heteropolymers by paired type‐I and type‐II keratin molecules in a context‐ and differentiation‐dependent manner.^13^ In the epidermis of normal skin, proliferating basal cells express K5 and K14,^14^ suprabasal cells synthesize K1 and K10. However, in certain pathologic conditions, an alternative pathway is open to KC, triggers KC activation.^15^ In this process, keratin expression alterations in the epidermis layers forming specific patterns, which is related to the significant modulation upon epidermal diseases,^10^, ^16^ the specific expression of keratin genes, K6/K16 and K17 have been used as markers for activated cells.^17^, ^18^, ^19^ In psoriasis, as the early barrier alarmins, over‐expressed K6/K16 and K17 alter the KCs behaviour, cell proliferation, adhesion, migration and inflammatory. KCs enter the state of hyperproliferation and innate immune activation, then trigger the T cells autoimmune activation, contributing the onset of psoriasis.^20^

We propose that KCs hyperproliferation and aberrant differentiation are contributing to the hard and thick lesional epidermis. Examining the profile of keratin expression in PN lesional epidermis provides a unique opportunity to understand the abnormal KC differentiation in PN. In this research work, to draw up the K1/K10, K5/K14, K6/K16 and K17 expression characterization, we investigated the keratin expression pattern in PN lesional skin using immunohistochemical staining.

## 3 METHODS

In this study, all the experiments were conducted in accordance with the ethical standards and approved by the ethical committee of the Peking University Shenzhen Hospital. All patients have signed informed consents.

### 3.1 Patient and specimen information

**FIGURE 1:**
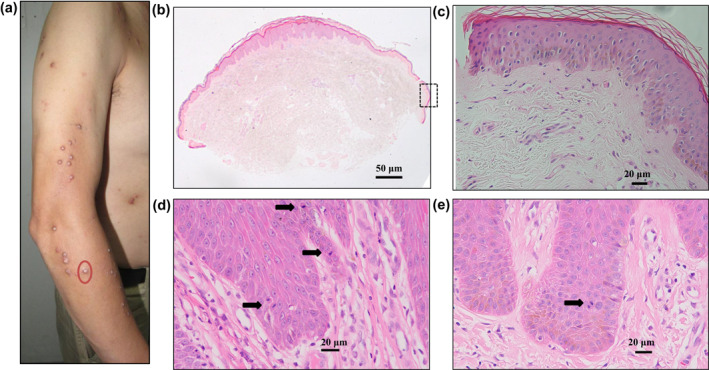
Histological study of lesional skin biopsy in patient with prurigo nodularis. (a), clinicopathological features of prurigo nodularis, nodules in circle was taken and divided into two parts, for mRNA analysis or histological experiments; Sections of skin biopsies were H&E stained; (b), histological observations of the lesional specimen in PN, original magnification: ×20; (c), perilesional epidermis, higher magnification of a region shown in the rectangular in (b), original magnification: ×200; increased mitotic activity cells within the basal layer (d) and suprabasal layer (e) in the lesions, arrowheads point the mitotic cells. Original magnification: ×400

PN patients diagnosis and questionnaire assess were described in our former paper.^21^ The lesional skin specimens were obtained from the extensor of the extremities of PN patients (Figure 1a and Table S1). Nine normal skin biopsies were taken from the arms, legs and trunk, when the volunteers underwent surgery for some other reasons.

### 3.2 H&E staining

According to standard procedures, all sections were accomplished the H&E staining for diagnosis and observation.

### 3.3 Morphologic analysis

Immunohistochemical staining analysis was conducted according to a previously described procedure.^8^, ^18^ Sections (4 μm) from paraffin‐embedded skin tissues were incubated with goat serum, then exposed to antibodies (Abs) (Table S2.) or control IgG. The following step was treated with one of procedures, (1) for immunofluorescence (IF), incubated with Alexa Fluor anti‐Rabbit (IgG) secondary antibody (Invitrogen), and stained with DAPI. (2) for Immunohistochemistry (IHC), incubated with secondary antibody peroxidase (HRP)‐labelled goat anti‐rabbit IgG (1:1) (Zhongshan Goldenbridge). Counterstained using a DAB kit (MXB, Biotechnologies).

All staining sections were detected using an epifluorescence microscopy (Zen, Carl Zeiss).

### 3.4 qRT‐PCR

The lesional and perilesional skin samples were extracted by TRIzol, according to the procedure described in the manufacturer's protocol (Sigma‐Aldrich), cDNA was obtained from the reverse transcription of RNA product by using a transcription kit (Vazyme Bioteck). Gene expression was quantified by qRT‐PCR protocol (Bio‐Rad, Hercules). Sequence‐specific primers were presented in the legend of Figure S3.

### 3.5 Statistical analysis

The relative keratin protein quantification method was performed according to the routines of Image J software (NIH). Gene expression differences between the lesional skin and normal healthy skin was done with GraphPad Prism 5 (GraphPad Software Inc.) as described in Zhong et al.^9^ SPSS 16.0 (SPSS Inc.) was used to calculate the spearman correlation among keratin gene, *p* ≤ 0.05 as statistically significant.

## 4 RESULT

### 4.1 Histology

All the biopsies were diagnosed by Routine H&E examination, confirming the clinical diagnosis with characteristic abnormal hyperkeratotic thick epidermis, focal parakeratosis, elongation on the rete ridges, and hypergranulosis in the epidermis (Figure 1a,b), compared with the surface of the normal healthy epidermis, which was relatively flat (Figure 1c).

### 4.2 Increased KC proliferation in basal and suprabasal layers

Epidermal rete ridges showed a folded structure with the convoluted basal layer, the enlargement of the basal layer resulted in greatly increased germinative cells. The basal layer cells in the lesional epidermal skin were closely packed with higher nuclear and cytoplasmic ratio, at the tip of rete ridges, more cells divided. In suprabasal layer, cell neuronal nuclei were enlarged with dense cytoplasm. Increased mitotic activity within the suprabasal layers showed the high proliferative status in the epidermis (Figure 1d,e,f). The increased numbers of epidermal KC piles up and the epidermis thickens.

## 5 KERATIN EXPRESSION

### 5.1 K5/K14 and K1/K10 expression profile in PN lesions

As keratins play essential roles in the KC, we were wondering whether the expression patterns of keratins would be changed during the lesional epidermis development. Immunohistochemical staining was used to detect the expression patterns of keratins. The staining results showed that K1, K10, K5 and K14 were all expressed in the border of the PN lesional nodular. In the border skin, K5 and K14 were expressed in basal cells, while differentiation cells in suprabasal layers express K1 and K10 (data not shown). These keratins expression pattern are almost same to the healthy control skin.

**FIGURE 2:**
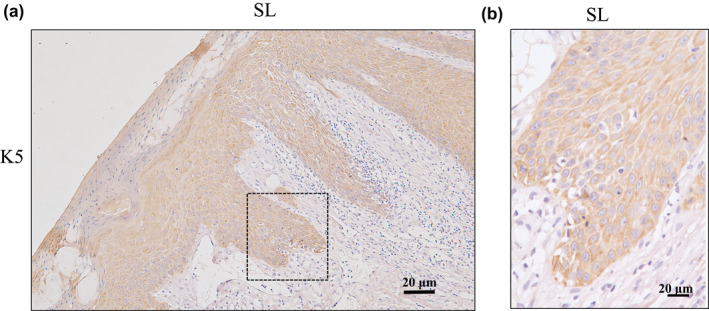
Immunohistochemical localization of K5 expression in the lesional skin epidermis. a, K5 signal in the lesional epidermis, b, is magnification of rectangle part in the Figure 2a, Original magnification : (a), ×100;(b), ×400. SL, lesional skin

In the PN lesional skin region, the K5 staining signal was detected in the basal layer and spinous layer, the signal was much stronger in the cells of basal layer and variable with an apparent gradient staining intensity towards suprabasal layer in the biopsies samples (Figure 2a,b). Furthermore, localization of K14 was the same as K5 in the PN lesional skin (Figure S2). In the staining signal positive KCs, the K5/K14 expression was observed in the cytoplasm, but not in the nuclei (Figure 2b and Figure S2).

**FIGURE 3:**
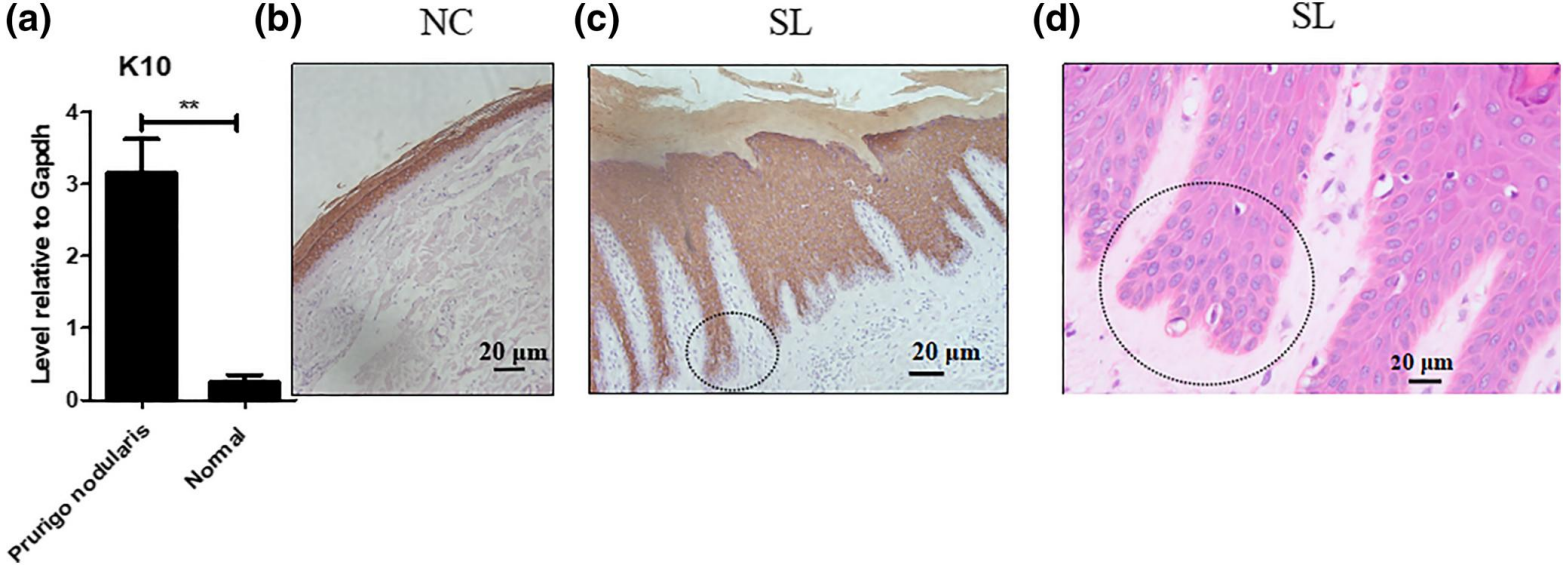
K10 expression in the PN lesional skin epidermis. (a), K10 protein levels comparison between lesional skin and normal healthy control by Western blot and Image J software analysis, **, *p* < 0.01; (b), K10 immunohistochemical localization in normal healthy control, original magnification: ×100; (c), K10 immunohistochemic al localization in PN lesions, original magnification: ×200; (d), H&E staining from the same biopsy in a neighbour slides, original magnification: ×400. Two blue circles in the (c) and (d) point the same location, which were sliced in the same specimen. NC, healthy control, SL, lesional skin

Compared with the normal skin, K10 protein expression level was higher in the lesional skin based on the western blot analysis (Figure 3a). K10 was detected with a strong signal in almost all the suprabasal layers cells. Except for the basal layer, K10 staining was completely spared even in the first and second suprabasal layers (Figure 3c), these cells were almost in the same cell morphology with bigger nuclei (Figure 3d).

For the PN lesions, K1 mRNA expression was highly correlated with K10 expression (*p* = 0.01) (Figure S3). K1 staining pattern was similar to the K10 in the PN lesional epidermis (Figure S3).

### 5.2 Hyperproliferation keratin expression

**FIGURE 4:**
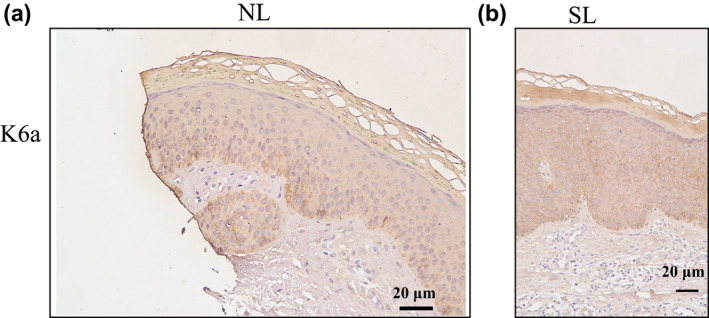
Immunohistochemical localization of K6a expression in the PN epidermis. Original magnification: (a), NL, ×200; (b), SL, ×100; NL, perilesional skin, SL, lesional skin

Staining KC labelled with K6 antibody was panepidermal in the PN lesional epidermis. We investigated two antibodies against K6a and K6b, they displayed similar expression profiles, which were presented in the differentiating suprabasal compartment of the interfollicular epidermis, especially in the spinous layer (Figure 4 and Figure S1). Different from the K6 localization, the staining pattern of K16 was limited in the basal layer and lower suprabasal layer cells (Figure S4).

K17 was induced in the lesional epidermis, it was detected throughout the cytoplasm of basal and suprabasal layer cells. The staining was patchy in the suprabasal layer, showing variable K17 expression levels (Figure S5).

## 6 DISCUSSION

The epidermis is a dynamic, continually renewing structure. In the normal epidermal skin, the germinative and post‐mitotic cells exist in the proliferative basal layer, they experience a transient amplification and divide into differentiated daughter cells. One of the daughter cells leaves the basal layer, move upwards to the epidermal surface, differentiate and form horny cell, ultimately, exfoliates.^22^, ^23^, ^24^

We observed mitotic cells in the suprabasal layers in the PN lesional skin (Figure 1d,e,f). Ki67 expression was localized in the lower suprabasal layers and basal layer cells (Figure S1), Ki67 is an antigen expressed in actively division cells, therefore Ki67 is used as a marker of cell proliferation.^25^ The increased mitotic activity within the basal and suprabasal layers thickens the spinous layer, the PN lesional epidermal architecture appears like amplified normal epidermal multilayers.

In the condition of injury and disease, keratin expression levels, localization or posttranslational modifications changed, consequently, keratin modulates cell migration, tumour growth et al.^10^ Therefore, keratin immunohistochemistry has been widely applied in surgical pathology for the differential diagnosis of undifferentiated malignancies since 1980s.^26^ To date, little is known about the keratin expression profile in PN lesional epidermis, and no research has been done to discover the possible correlation of KC proliferation and differentiation with the keratin expression.

KC in PN lesional skin become abnormally activated, K5/K14 strongly positive staining signal distributed in basal and spinous layer cells, which were consistent with increased mitotic activity within the suprabasal layer (Figure 1d,e). K6/K16 and K17 mRNA levels in the PN lesional skin were found to be significantly higher than the perilesional skin.^20^ K16 and K17 expression are limited in the lower suprabasal layer and basal layers (Figures S4 and S5). Their expression patterns in PN epidermis are quite different from the K6 (K6a and K6b), they are over‐expressed throughout the interfollicular epidermis in PN nodularis. K6 is required to the expression of desmoplakin, which connect the intermediate filaments and desmosome to regulate the KC cell‐cell and cell‐matrix adhesion.^27^ We speculate that K6 is binding with another protein to form complex in the upperbasal layers, and play an important role in the tight and compact epidermis forming, which need to be proved in the future.

In the PN lesional skin epidermis, the K1 and K10 are over‐expressed in the panepidermis, expression levels were increased (Figure 3c and Figure S3). In contrast to the psoriasis lesions, K1/K10 levels are significantly reduced, which are interpreted as a defect of terminal cell differentiation. These results are consistent with the histological observation between PN and psoriasis.^28^, ^29^

The keratin expression profile in PN epidermis was summarized (Table S3), they are different from the healthy epidermis, the psoriasis,^30^, ^31^ hypertrophic scar^11^ and squamous cell carcinoma,^32^ In a summary, hyperproliferative and aberrant differentiation of KCs are involved in the hard cornification in the thick epidermis development,^6^, ^21^, ^33^ it offers a deeper insight to understand epidermal hyperplasia and PN pathogenesis.

K5 and K6 are encoded by neighbouring genes, they are paralogs in the keratin cluster analysis, so do the K14, K16 and K17.^10^ Previous studies proved that the activation process can be affected by growth factors and cytokines (e.g. IL‐1, TNF‐α, TGF‐α, TGF‐β and IFN‐γ).^34^ Recent research discovered the upregulation of epidermal inflammatory ILs (IL‐4 and IL‐17), epidermal receptor expression, MAPK signalling^9^ in the PN lesional skin, which is helpful to investigate how the keratins are regulated in the KC in the lesions.

In psoriasis, K17 is a potential therapeutic target, K17‐specific antisense oligonucleotides could knock down the K17 protein content and inhibit the KC proliferation in vitro.^28^ In clinical practice, IL‐17A antibodies (Secukinumab and Ixekizumab), and IL‐17 receptor A inhibitors (Brodalumab) could reduce the K17 expression,^35^ which are effective and safe therapies for psoriasis. The keratinizing disorder of pathyonychia congenital is mainly caused by the mutation in K6, K16 and K17 genes, the siRNA for the site of K6aN171K mutant is a clinical efficacy treatment.^36^ These treatments prompt us important clues in the therapy for PN.

## 7 CONCLUSIONS

Finally, epidermal KC may play an important role in the pathogenesis of the PN, we focused on the relationship between the proliferation of abnormal KC in PN skin lesions and keratin expression patterns. Hyperproliferative and aberrant differentiation of KCs are involved in the epidermal hyperplasia and orthohyperkeratosis. Keratins play vital roles in the PN lesion forming process, their expression data help us to understand the clinical and morphological characters in the PN lesions. In the future, the potential signalling regulation system and over‐expressed keratins are going to be new treatment targets for the PN patients.

## CONFLICT OF INTEREST

No conflicts of interest to declare.

## AUTHOR CONTRIBUTIONS

**L. L. Yang:** Funding acquisition; Investigation; Methodology; Software; Writing – original draft; Writing – review & editing. **B. Jiang:** Investigation; Resources; Software; Supervision. **S. H. Chen:** Investigation; Methodology; Resources; Supervision; Validation. **T. T. Chen:** Methodology; Resources. **L. H. Huang:** Investigation; Methodology; Resources. **M. Yang:** Investigation; Methodology; Project administration; Resources. **J. Ding:** Methodology; Resources. **J. J. He:** Data curation; Methodology; Project administration; Resources; Supervision. **J. J. Li:** Conceptualization; Data curation; Investigation; Project administration; Resources; Supervision; Writing – review & editing. **B. Yu:** Conceptualization; Funding acquisition; Methodology; Project administration; Resources; Supervision; Writing – review & editing.

## Supporting information

Table S1

Table S2

Table S3

Figure S1

Figure S2

Figure S3

Figure S4

Figure S5

## Data Availability

The data that support the findings of this study are available from the corresponding author upon reasonable request.

## References

[1] Zeidler C , Tsianakas A , Pereira M , Ständer H , Yosipovitch G , Ständer S . Chronic prurigo of nodular type: a review. Acta Derm Venereol. 2018;98(2):173–9. 29135018 10.2340/00015555-2774

[2] Schedel F , Schūrmann C , Metze D , Ständer S . [Prurigo. Clinical definition and classification]. Hautarzt. 2014;65(8):684–90. 25113326 10.1007/s00105-014-2753-z

[3] Kwon CD , Khanna R , Williams KA , Kwatra MM , Kwatra SG . Diagnostic workup and evaluation of patients with prurigo nodularis. Medicines (Basel). 2019;6(4):97. 10.3390/medicines6040097 PMC6963711 31561504

[4] Kowalski EH , Kneiber D , Valdebran M , Patel U , Amber KT . Treatment‐resistant prurigo nodularis: challenges and solutions. Clin Cosmet Invest Dermatol. 2019;12:163–72. 10.2147/CCID.S188070 PMC6400231 30881076

[5] Zeidler C , Yosipovitch G , Ständer S . Prurigo nodularis and its management. Dermatol Clin. 2018;36(3):189–97. 29929592 10.1016/j.det.2018.02.003

[6] Zeidler C , Ständer S . The pathogenesis of prurigo nodularis‐‐'super‐itch' in exploration. Eur J Pain. 2016;20(1):37–40. 26416433 10.1002/ejp.767

[7] Ständer S , Pereira M , Berger T , Zeidler C , Augustin M , Bobko S , et al. IFSI‐guideline on chronic prurigo including prurigo nodularis. J Itch. 2020;5(4):e42.

[8] Zhong W , Wu X , Zhang W , Zhang J , Chen X , Chen S , et al. Aberrant expression of histamine‐independent pruritogenic mediators in keratinocytes may be involved in the pathogenesis of prurigo nodularis. Acta Derm Venereol. 2019;99(6):579–86. 30809683 10.2340/00015555-3150

[9] Agelopoulos K , Rülander F , Dangelmaier J , Lotts T , Osada N , Metze D , et al. Neurokinin 1 receptor antagonists exhibit peripheral effects in prurigo nodularis including reduced ERK1/2 activation. J Eur Acad Dermatol Venereol. 2019;33(12):2371–9. 31442331 10.1111/jdv.15905

[10] Toivola DM , Boor P , Alam C , Strnad P . Keratins in health and disease. Curr Opin Cell Biol. 2015;32:73–81. 25599598 10.1016/j.ceb.2014.12.008

[11] Machesney M , Tidman N , Waseem A , Kirby L , Leigh I . Activated keratinocytes in the epidermis of hypertrophic scars. Am J Pathol. 1998;152(5):1133–41. 9588880 PMC1858601

[12] Moll R , Franke WW , Schiller DL , Geiger B , Krepler R . The catalog of human cytokeratins: patterns of expression in normal epithelia, tumors and cultured cells. Cell. 1982;31(1):11–24. 6186379 10.1016/0092-8674(82)90400-7

[13] Steinert PM . The two‐chain coiled‐coil molecule of native epidermal keratin intermediate filaments is a type I‐type II heterodimer. J Biol Chem. 1990;265(15):8766–74. 1692836

[14] Sun TT , Eichner R , Schermer A . Cooper D , Nelson WG , Weiss RA . The transformed phenotype. Classification, expression, and possible mechanisms of evolution of mammalian epithelial keratins: a unifying model. 2nd ed., Vol. 1. New York: Cold Spring Harber laboratory; 1984. p.169–176.

[15] Barker JN , Mitra RS , Griffiths CE , Dixit VM , Nickoloff BJ , Nickoloff BJ . Keratinocytes as initiators of inflammation. Lancet. 1991;337(8735):211–14. 1670850 10.1016/0140-6736(91)92168-2

[16] Moll R , Divo M , Langbein L . The human keratins: biology and pathology. Histochem Cell Biol. 2008;129(6):705–33. 18461349 10.1007/s00418-008-0435-6 PMC2386534

[17] Mockler D , Escobar‐Hoyos LF , Akalin A , Romeiser J , Shroyer AL , Shroyer KR . Keratin 17 is a prognostic biomarker in endocervical glandular neoplasia. Am J Clin Pathol. 2017;148(3):264–73. 28821199 10.1093/ajcp/aqx077

[18] Wang F , Zieman A , Coulombe PA . Skin keratins. Methods Enzymol. 2016;568:303–50. 26795476 10.1016/bs.mie.2015.09.032 PMC4902878

[19] Hu H , Xu DH , Huang XX , Zhu CC , Xu J , Zhang ZZ , et al. Keratin17 promotes tumor growth and is associated with poor prognosis in gastric cancer. J Cancer. 2018;9(2):346–57. 29344281 10.7150/jca.19838 PMC5771342

[20] Zhang X , Yin M , Zhang LJ . Keratin 6, 16 and 17‐critical barrier alarmin molecules in skin wounds and psoriasis. Cells. 2019;8(8). 10.3390/cells8080807 PMC6721482 31374826

[21] Yang LL , Huang HY , Chen ZZ , Chen R , Ye R , Zhang W , et al. Keratin 17 is induced in prurigo nodularis lesions. Open Chemistry. 2020;18(1):463–71.

[22] Eckert RL , Crish JF , Robinson NA . The epidermal keratinocyte as a model for the study of gene regulation and cell differentiation. Physiol Rev. 1997;77(2):397–424. 9114819 10.1152/physrev.1997.77.2.397

[23] Iizuka H , Takahashi H , Ishida‐Yamamoto A . Psoriatic architecture constructed by epidermal remodeling. J Dermatol Sci. 2004;35(2):93–9. 15265521 10.1016/j.jdermsci.2004.01.003

[24] Jones PH , Harper S , Watt FM . Stem cell patterning and fate in human epidermis. Cell. 1995;80(1):83–93. 7813021 10.1016/0092-8674(95)90453-0

[25] Gerdes J , Lemke H , Baisch H , Wacker HH , Schwab U , Stein H . Cell cycle analysis of a cell proliferation‐associated human nuclear antigen defined by the monoclonal antibody Ki‐67. J Immunol. 1984;133(4):1710–15. 6206131

[26] Corson JM . Keratin protein immunohistochemistry in surgical pathology practice. Pathol Annu. 1986;21:47–81. 2427996

[27] Wang F , Chen S , Liu HB , Parent CA , Coulombe PA . Keratin 6 regulates collective keratinocyte migration by altering cell‐cell and cell‐matrix adhesion. J Cell Biol. 2018;217(12):4314–30. 30389720 10.1083/jcb.201712130 PMC6279382

[28] Chang T , Sun L , Wang Y , Wang D , Li W , Li C. Inhibition of keratin 17 expression with antisense and RNAi strategies: exploring novel therapy for psoriasis. Exp Dermatol. 2011;20(7):555–60. 21355890 10.1111/j.1600-0625.2010.01235.x

[29] Mazzalupo S , Wong P , Martin P , Coulombe PA . Role for keratins 6 and 17 during wound closure in embryonic mouse skin. Dev Dynam. 2003;226(2):356–65. 10.1002/dvdy.10245 12557214

[30] Fu M , Wang G . Keratin 17 as a therapeutic target for the treatment of psoriasis. J Dermatol Sci. 2012;67(3):161–5. 22795618 10.1016/j.jdermsci.2012.06.008

[31] Thewes M , Stadler R , Korge B , Mischke D . Normal psoriatic epidermis expression of hyperproliferation‐associated keratins. Arch Dermatol Res. 1991;283(7):465–71. 1724897 10.1007/BF00371784

[32] Proby CM , Churchill L , Purkis PE , Glover MT , Sexton CJ , Leigh IM . Keratin 17 expression as a marker for epithelial transformation in viral warts. Am J Pathol. 1993;143(6):1667–78. 7504888 PMC1887274

[33] Weigelt N , Metze D , Ständer S . Prurigo nodularis: systematic analysis of 58 histological criteria in 136 patients. J Cutan Pathol. 2010;37(5):578–86. 20002240 10.1111/j.1600-0560.2009.01484.x

[34] Freedberg IM , Tomic‐Canic M , Komine M , Blumenberg M . Keratins and the keratinocyte activation cycle. J Invest Dermatol. 2001;116(5):633–40. 11348449 10.1046/j.1523-1747.2001.01327.x

[35] Conrad C , Gilliet M . Psoriasis: from pathogenesis to targeted therapies. Clin Rev Allergy Immunol. 2018;54(1):102–13. 29349534 10.1007/s12016-018-8668-1

[36] Leachman SA , Hickerson RP , Schwartz ME , Bullough EE , Hutcherson SL , Boucher KM , et al. First‐in‐human mutation‐targeted siRNA phase Ib trial of an inherited skin disorder. Mol Ther. 2010;18(2):442–6. 19935778 10.1038/mt.2009.273 PMC2839285

