# Supplementary material for: Abnormal keratin expression pattern in prurigo nodularis epidermis
Source: Skin Health Dis. 2021 Dec 1;2(1):e75. doi: 10.1002/ski2.75 (PMC9060049; doi:10.1002/ski2.75)
Supplement: Supplementary file 1 — Table S1 [file SKI2-2-e75-s001.docx]

| Number of Patient | Biopsy site | Average age | Sex |
| --- | --- | --- | --- |
| 6 | arm | 43 | F:M(4:2) |
| 6 | limb | 48.8 | F:M(3:3) |
| 12 | Truck | 49 | F:M(4:8) |

Suppl. table 1. Source of prurigo nodularis biopsy in this study.
