# Supplementary material for: Abnormal keratin expression pattern in prurigo nodularis epidermis
Source: Skin Health Dis. 2021 Dec 1;2(1):e75. doi: 10.1002/ski2.75 (PMC9060049; doi:10.1002/ski2.75)
Supplement: Supplementary file 2 — Table S2 [file SKI2-2-e75-s004.docx]

Supplementary table 2. The antibodies and optimized antigen retrieval methods for immunohistochemical staining.

| Antibody | Source | Host Species | Clonality | Antibody  dilution | Antigen  retrieval |
| --- | --- | --- | --- | --- | --- |
| Keratin 1 | Abcam  (ab185628) | Rabbit | Monoclonal | 1:600 | Tris/EDTA |
| Keratin 5 | Abcam  （ab52635） | Rabbit | Monoclonal | 1:200 | Sodium Citrate |
| Keratin 6 | Abcam  (ab18586) | Mouse | Monoclonal | 1:500 | Sodium Citrate |
| Keratin 6a | GeneTex  (GXT 105355） | Rabbit | Polyclonal | 1:500 | Sodium Citrate |
| Keratin 6b | GeneTex  (GXT 110426） | Rabbit | Polyclonal | 1:300 | Sodium Citrate |
| Keratin10 | Abcam  (ab9025) | Mouse | Monoclonal | 1:200 | Sodium Citrate buffer |
| Keratin10 | GeneTex  (GTX21421) | Mouse | Monoclonal | 1:200 | Sodium Citrate buffer |
| Ketatin14 | Abcam  (ab181595) | Rabbit | Monoclonal | 1:200 | Sodium Citrate buffer |
| Keratin16 | Abcam  (ab76416) | Rabbit | Monoclonal | 1:100 | EDTA buffer |
| Keratin16 | GeneTex  (GTX100838) | Rabbit | Polyclonal | 1:100 | EDTA based |
| Keratin17 | GeneTex  (GTX1) | Rabbit | Polyclonal | 1:100 | Sodium Citrate |
| Keratin17 | Abcam  (Ab109725) | Rabbit | Monoclonal | 1:100 | Sodium Citrate |
| Ki-67 | Cell Signaling Technology  Cst#9449 | Mouse | Monoclonal | 1:400 | Sodium Citrate |
