# Supplementary material for: Abnormal keratin expression pattern in prurigo nodularis epidermis
Source: Skin Health Dis. 2021 Dec 1;2(1):e75. doi: 10.1002/ski2.75 (PMC9060049; doi:10.1002/ski2.75)
Supplement: Supplementary file 3 — Table S3 [file SKI2-2-e75-s002.docx]

Supplementary table 3. Summary results of immunohistochemistry for keratin in prurigo nodularis lesional epidermis.

| Antibody | Prurigo nodularis | Psoriasis[[1](#_ENREF_1), [2](#_ENREF_2)] | Normal skin |
| --- | --- | --- | --- |
| K1 | SL and GL | SB | SL and GL |
| K5 | BL and SL | BL | BL |
| K6 | BL,SL and GL | Interfollicular SB | N |
| K10 | SL and SB | SB | SL and GL |
| K14 | BLand SL | BL | BL |
| K16 | BL and LSB | Interfollicular SB | N |
| K17 | BLand SL | Interfollicular SB | N |

The epidermal location is compared with the reported for psoriasis and normal skin.

Abbreviations: BL, basal layer; GL, granular layer; SL, spinous layer; SB, suprabasal layer; USB, upper suprabasal layer; LSB, lower suprabasal layer.
