# Supplementary figures and images for: Abnormal keratin expression pattern in prurigo nodularis epidermis
Source: Skin Health Dis. 2021 Dec 1;2(1):e75. doi: 10.1002/ski2.75 (PMC9060049; doi:10.1002/ski2.75)

## Supplementary Figure 2. K14 immunohistochemical location in the lesional skin.

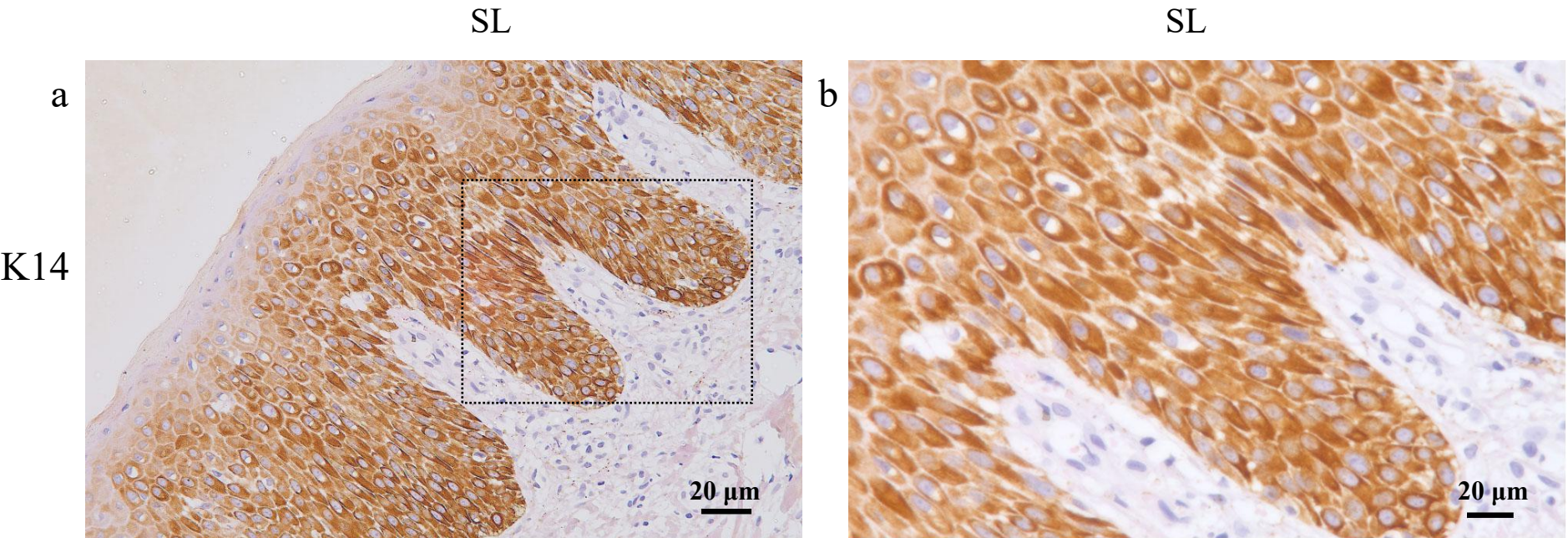

Supplement: Supplementary file 5 — Figure S2 [file SKI2-2-e75-s007.pdf]

# Supplementary Figure 3. K1 expression in the lesional skin

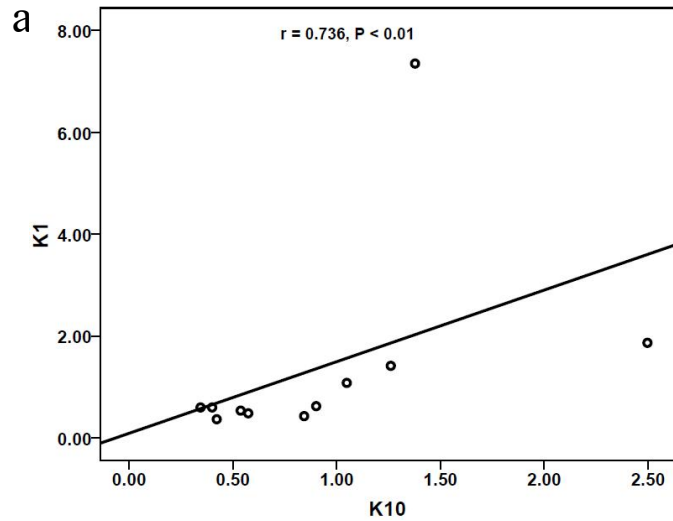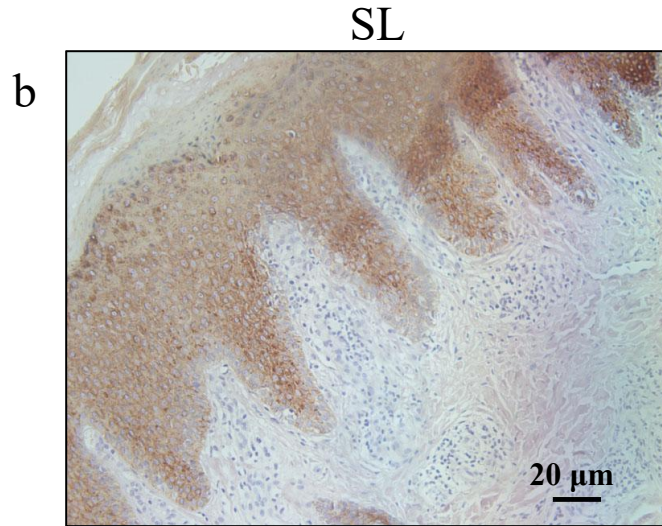

Supplement: Supplementary file 6 — Figure S3 [file SKI2-2-e75-s006.pdf]
