# Supplementary material for: Abnormal keratin expression pattern in prurigo nodularis epidermis
Source: Skin Health Dis. 2021 Dec 1;2(1):e75. doi: 10.1002/ski2.75 (PMC9060049; doi:10.1002/ski2.75)

Supplementary Figure 4. Immunohistochemical localization of K16 expression in the PN lesional epidermis

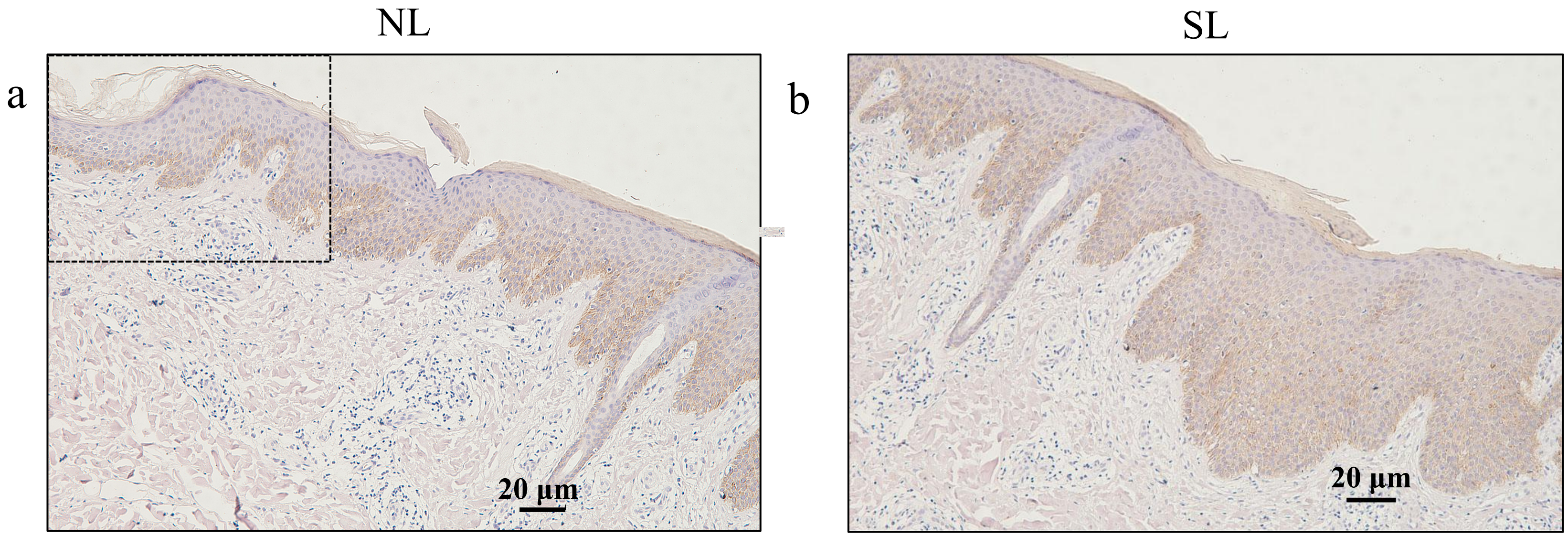

Supplement: Supplementary file 7 — Figure S4 [file SKI2-2-e75-s005.pdf]
