# Supplementary material for: Abnormal keratin expression pattern in prurigo nodularis epidermis
Source: Skin Health Dis. 2021 Dec 1;2(1):e75. doi: 10.1002/ski2.75 (PMC9060049; doi:10.1002/ski2.75)

Supplementary Figure 5. Immunohistochemical localization of K17 expression in the lesional and perilesional skin

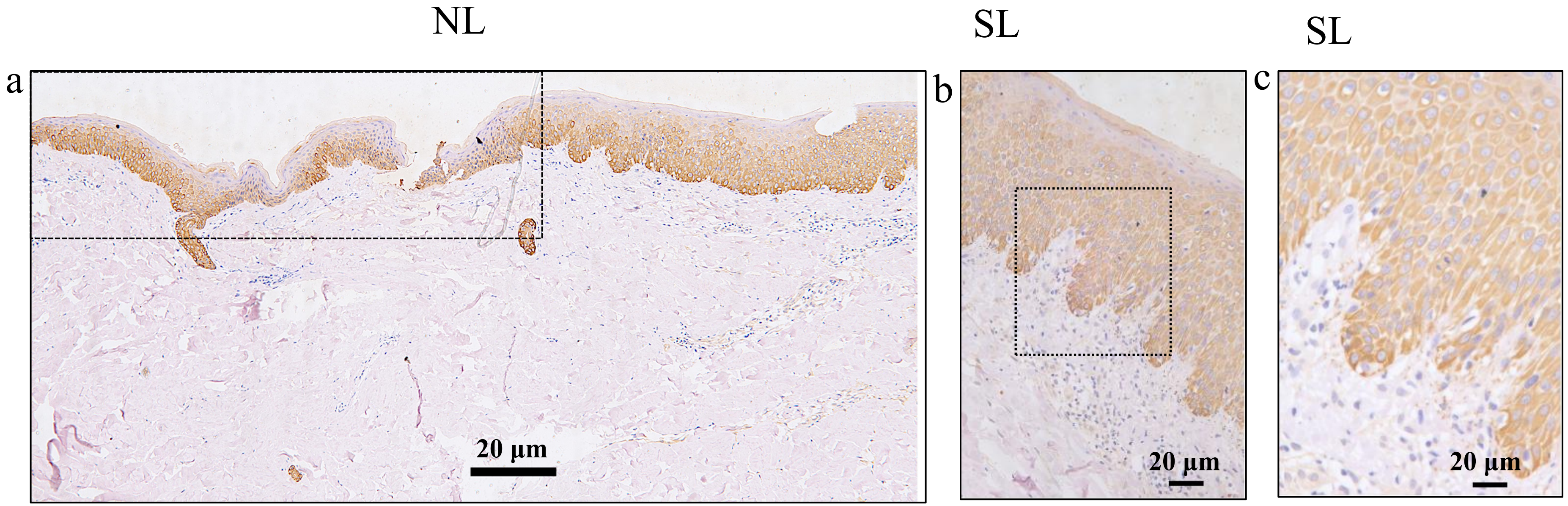

Supplement: Supplementary file 8 — Figure S5 [file SKI2-2-e75-s008.pdf]
